# Environmental sustainable ZrO_2_ -phosphorous Biochar nano composite derived from sugarcane bagasse and their adsorption behavior of antidepressant drugs

**DOI:** 10.1186/s13065-025-01430-4

**Published:** 2025-03-14

**Authors:** Walaa A. Elhamdy

**Affiliations:** Chemistry Department, Faculty of Science, Sohag University, P.O. Box 82524, Sohag, Egypt

**Keywords:** Biochar, Zirconia, Antidepressant pollution, Adsorption mechanism

## Abstract

Phosphorous biochar was synthesized from sugarcane bagasse (SB) by applying a 2:1 weight ratio of H_3_PO_4_ to OP and pyrolyzing it at 600 °C under nitrogen. Sugarcane bagasse was selected for its affordability and environmental benefits as a carbon support. Following this, a zirconium-loaded PC nanocomposite (ZrP400) was developed by impregnating zirconium hydroxide in concentrations 5–30% onto the mesoporous phosphorous biochar, which was then thermally treated at 400ºC. Analytical techniques showed that the ZrP400 adsorbents had a high surface area (1697–2434 m²/g) and considerable porosity. The effectiveness of these adsorbents in removing the hazardous tricyclic antidepressant amitriptyline (AMT) from water was tested. At a pH of 6.52, the neutral adsorbent provided various chemical functional groups that facilitated the binding of amitriptyline. With 20 mg of adsorbent at 35ºC, the capacity for amitriptyline adsorption reached up to 585 mg/g. Adsorption equilibrium was reached within 120 min over a concentration range of 10 to 300 mg/L. Kinetic and equilibrium data showed that the adsorption was well described by the pseudo-second-order and Freundlich isotherm models, indicating that chemisorption was the primary mechanism, with physisorption also contributing significantly to amitriptyline removal. The spent adsorbent could be effectively regenerated using ethanol. Additionally, the process’s sustainability was assessed using GAPI and AGREE metrics, which confirmed its environmental friendliness, practicality, and sustainability.

**Graphical Abstract:**

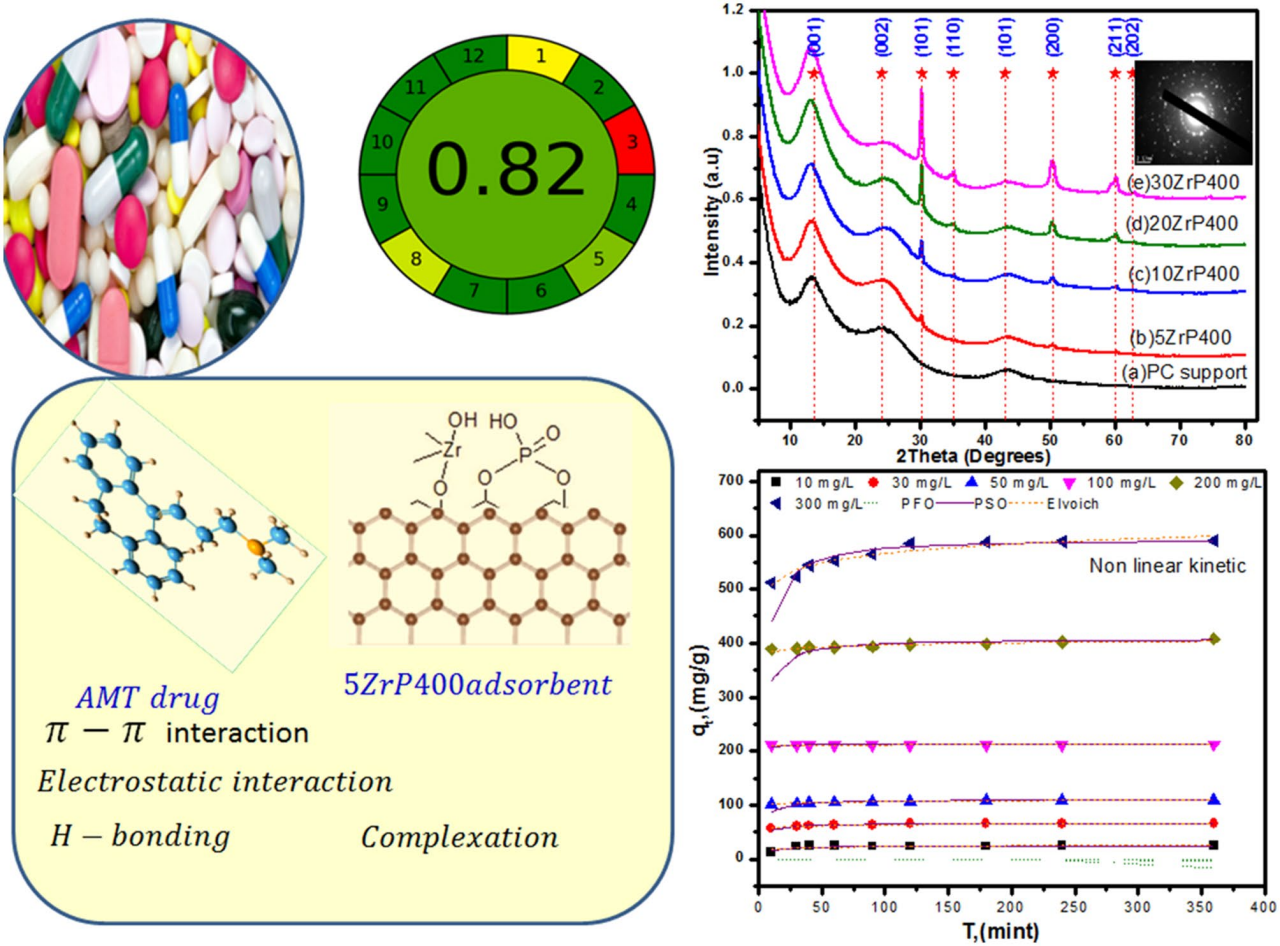

**Supplementary Information:**

The online version contains supplementary material available at 10.1186/s13065-025-01430-4.

## Introduction

Globally, approximately 0.3 billion people suffer from depression daily, making it the most common mental health disorder in modern society. Alarmingly, depression is responsible for nearly 0.8 million suicide deaths each year, ranking as the leading cause of mental disability worldwide [1]. The COVID-19 pandemic has further exacerbated this issue, leading to a notable rise in anxiety and depression cases globally [2]. Amitriptyline, a commonly prescribed tricyclic antidepressant (TCA), is widely used to alleviate stress and regulate behavioral imbalances. Its therapeutic effects are partially attributed to its inhibitory action on receptors such as histamine, muscarinic, and post-synaptic alpha-adrenergic receptors [3–5]. However, residues of amitriptyline, reported at levels up to 900 ng/L in treated effluent [6], persist in the aquatic environment due to the incomplete removal of pharmaceuticals in wastewater treatment plants. These residues pose significant environmental risks, underscoring the need for effective and eco-friendly methods to remove such contaminants and restore the balance between human activities and the environment.

A range of techniques has been employed for wastewater treatment, including filtration, reverse osmosis, ultrafiltration, solvent extraction, coagulation, advanced oxidation, microbial reduction, and adsorption [7, 8]. Among these, adsorption has emerged as a highly effective method due to its sensitivity to micropollutants, cost efficiency, and environmental compatibility [9].

Recent advancements have introduced a variety of adsorbents, such as metal-organic frameworks [10], activatedcarbon [11], zeolites [12], biomasses [13–15], and graphene-based porouscarbon [16]. Biomass serves as a readily accessible, sustainable, and cost-effective precursor for biochars which are widely used as adsorbents to remove pollutants utilized in energy and environmental sectors [17–30]. Biochar, a carbon-rich material produced through the pyrolysis of biomass under oxygen-limited conditions [31], holds significant potential for various applications. These include enhancing crop yields [32], purifying water [33, 34], remediating pollutants in soil and sediment [35, 36], producing bioenergy [37], and sequestering carbon [38]. Notably, biochar has shown great promise in effectively removing antibiotics and pharmaceutical drugs due to its abundant pores and rich functional groups [39, 40].

Functionalized biochar, tailored through surface modifications to enhance adsorption sites, demonstrates increased efficiency in removing specific pollutants [41–48]. Additionally, different types of biomass naturally contain elements such as nitrogen, oxygen, potassium and phosphorous, which can function as doped heteroatoms on carbon surfaces, potentially activating them in the process. Numerous research studies have explored the production of biochar from agricultural waste and their effectiveness in eliminating emerging contaminants. Natural materials, especially agro-waste, are gaining considerable attention as key raw materials for producing active substances due to their economic, environmental, and technological advantages. Globally, around 700 million tons of sugarcane bagasse is generated annually [49]. Brazil, as the leading producer of sugarcane, contributes more than 625 million tons to this total [50], with approximately 280 kg of bagasse produced per ton of sugarcane processed [51]. Despite its significant potential for a variety of applications, the current utilization of sugarcane bagasse remains limited, and its full economic value is yet to be fully realized [52].

Sugarcane bagasse derived biochar has been investigated for its effectiveness in adsorbing various pollutants, such as antibiotics [53], phenol [54], and metal ions [55]. It has been demonstrated that zirconia has shown promise in a variety of sectors, including electrostatic coatings, refractory materials, ceramic materials, and catalysts [56]. Due to its many advantages, including strong affinity for electronegative ligands, superior biocompatibility, acid-base stability, ease of modification, low cost, and non-toxicity, zirconia has emerged as a highly competitive material for adsorbents today. Numerous types of adsorbents based on zirconia have been used to treat water pollution [57].

Zirconia, with its strong affinity for electronegative ligands, high biocompatibility, and ease of modification, has proven to be an excellent material for adsorbent applications. Zirconia-based composites have been extensively studied for treating water pollution, with notable examples including Co₃O₄-ZrO₂ for dye adsorption [58] CaO-doped tetragonal ZrO_2_ [59] for adsorption of organic dyes. Building on this foundation, the present study investigates the synthesis of phosphorus biochar from sugarcane bagasse and the development of zirconia-supported phosphorus biochar (ZrP) adsorbents. The primary aim is to characterize these materials using techniques such as XRD, FTIR, XPS, TEM, and BET analysis, and evaluate their performance in removing amitriptyline from water. This research focuses on correlating the structural and textural properties of ZrP biochar with its adsorption capabilities. Removal efficiencies and adsorption capacities under various conditions will be assessed, along with kinetic, adsorption, and thermodynamic analyses to elucidate the removal mechanism. Specifically, the study will highlight the supported zirconia ZrP400 adsorbent, providing insights into its potential as an effective and sustainable solution for pharmaceutical wastewater treatment.

## Experimental sections

### Chemicals

In this study, sugarcane bagasse residue was used as a raw material, sourced from Egypt during the peak of the season. Bagasse is obtained from sugar mills as a byproduct of sugarcane processing in Sohag region, in Upper Egypt. Phosphoric acid (H₃PO₄, 85%), hydrochloric acid (HCl, 36%), and sodium hydroxide (NaOH) were obtained from Alpha (Egypt). Zirconium hydroxide (Zr(OH)_4_) was purchased from Sigma-Aldrich (Germany). Amitriptyline was provided by Rameda Co. for Pharmaceutical Industries & Diagnostic Reagents (6th of October City, Egypt).

### ZrO_2_-embedded phosphorus Biochar nano composite fabrication

Sugarcane bagasss were rinsed with deionized water, dried at 120 °C for 12 h, and ground to 100 mesh. The ground sugarcane bagasss were mixed with 85% phosphoric acid (H_3_PO_4_) in a 2:1 (w/w) ratio. The mixture was sonicated for 0.5 h, and then mechanically stirred for 1 h. The mixture was allowed to stand for 24 h. The mixture was heated at 110 °C for 24 h. The material was then activated at 600 °C for 1 h under N_2_ flow, with a ramp rate of 10 °C/min. The activated material was repeatedly rinsed with 1 M HCl and hot/cold distilled water until the pH reached 6.5.The material was then dried overnight at 120 °C.The phosphoric-acid-activated carbon obtained is referred to as PC sample. Zirconium oxide-embedded phosphoric biochar (ZrP400) was synthesized by impregnating the PC sample with 5–30% (w/w) aqueous zirconium hydroxide solution. The impregnated material was dried at 120 °C for 24 h. The dried material was then thermally treated at 400 °C for 3 h under N_2_ atmosphere. The treated carbon was separated using a 100-mesh filter. Various characterization techniques were used, including XRD, FTIR-ATR, HRTEM, EDX, FSEM, BET, XPS, and UV-vis spectrophotometry.

### AMT adsorption test

Using an automated orbiting shaker, batch adsorption studies were carried out to evaluate the adsorption of a medication called AMT onto the PC and ZrP400 adsorbents. Testing the initial pH of the adsorption solution (which ranges from 2 to 12) and modifying the pH with 0.1 M NaOH and/or 0.1 M HCl allowed experts to identify the point of zero charge (pHpzc) of the ZrP400 material’s surface. UV spectrophotometry at 239 nm was used to measure the AMT concentrations prior to (C_o_) and following (C_e_) adsorption. By employing Eqs. (1) and (2):

1 $$\:{\:\:\:\:q}_{e\:=\:\frac{\left({C}_{o\:-\:}{C}_{e}\right)}{m}}v$$

2 $$\:\text{E}\text{f}\text{f}\text{i}\text{c}\text{i}\text{e}\text{n}\text{c}\text{y}\:\text{o}\text{f}\:\text{r}\text{e}\text{m}\text{o}\text{v}\text{a}\text{l}\:\:\left(\%\right)=\frac{{c}_{o}-{c}_{e}}{{c}_{o}}\times\:100$$

The adsorption capacity is denoted by q_e_ (mg/g); the solution volume (L) is represented by V; the adsorbent mass (g) is represented by m; and the initial and equilibrium concentrations of AMT are indicated by C_0_ and C_e_ (mg L^− 1^).

Adsorption equilibrium times up to 24 h were studied, with samples taken at regular intervals to measure the AMT concentration in the filtrate until equilibrium was reached. Initial AMT concentrations ranging from 10 to 300 mg/L were examined. The time-dependent adsorption capacity (q_t_) was calculated using the equation.

3 $$\:{\:\:\:\:q}_{t\:=\:\frac{\left({C}_{o\:-\:}{C}_{t}\right)}{m}}v$$

The effect of adsorption temperature (35–55 °C) was studied for 100 and 200 mg/L AMT concentrations. Thermodynamic parameters were calculated using the Van’t Hoff equation. Eqs. (4) and (5).

4 $$\:\:\:\:\:\:\:\:\:\:\:\:\:\varDelta\:{G}^{o}=\varDelta\:{H}^{o}-T\varDelta\:{S}^{o}$$

5 $$\:\text{ln}{K}_{c}=\frac{{\Delta\:}\text{S}^\circ\:\:}{R}-\frac{{\Delta\:}\text{H}^\circ\:}{R}\left(\frac{1}{T}\right)$$

Where T is the absolute temperature of solution (*K*), R denotes the universal gas constant, K_C_ is the adsorption equilibrium constant, *ΔG°* is the standard enthalpy change (*ΔH°*), and *ΔS°* is the thermodynamic parameter of the adsorbent.

To desorb the adsorbed AMT and preserve the reusability of the ZrP400 adsorbent, the following procedure was used: The ZrP400 adsorbent was shaken for 60 min at 35 ± 1 °C with ethanol. The mixture was then filtered to recover the desorbed AMT eluent. Using new eluent, desorption procedure was carried on many times until no further AMT desorption was seen. The ZrP400 adsorbent’s reusability (%) was calculated with the help of the following formula: Eq. (6)

6 $$\:\:\:reusability\%=\frac{{C}_{o}\:-{C}_{e}\:}{{C}_{d}}\:\times\:100$$

The desorption AMT concentration is represented by C_d_ (mg/l), while the initial and equilibrium concentrations are represented by C_0_ and C_e_ (mg/L), respectively.

## Results and discussion

### Texture and surface characteristics of the synthetic carbon nano composite

**Fig. 1:**
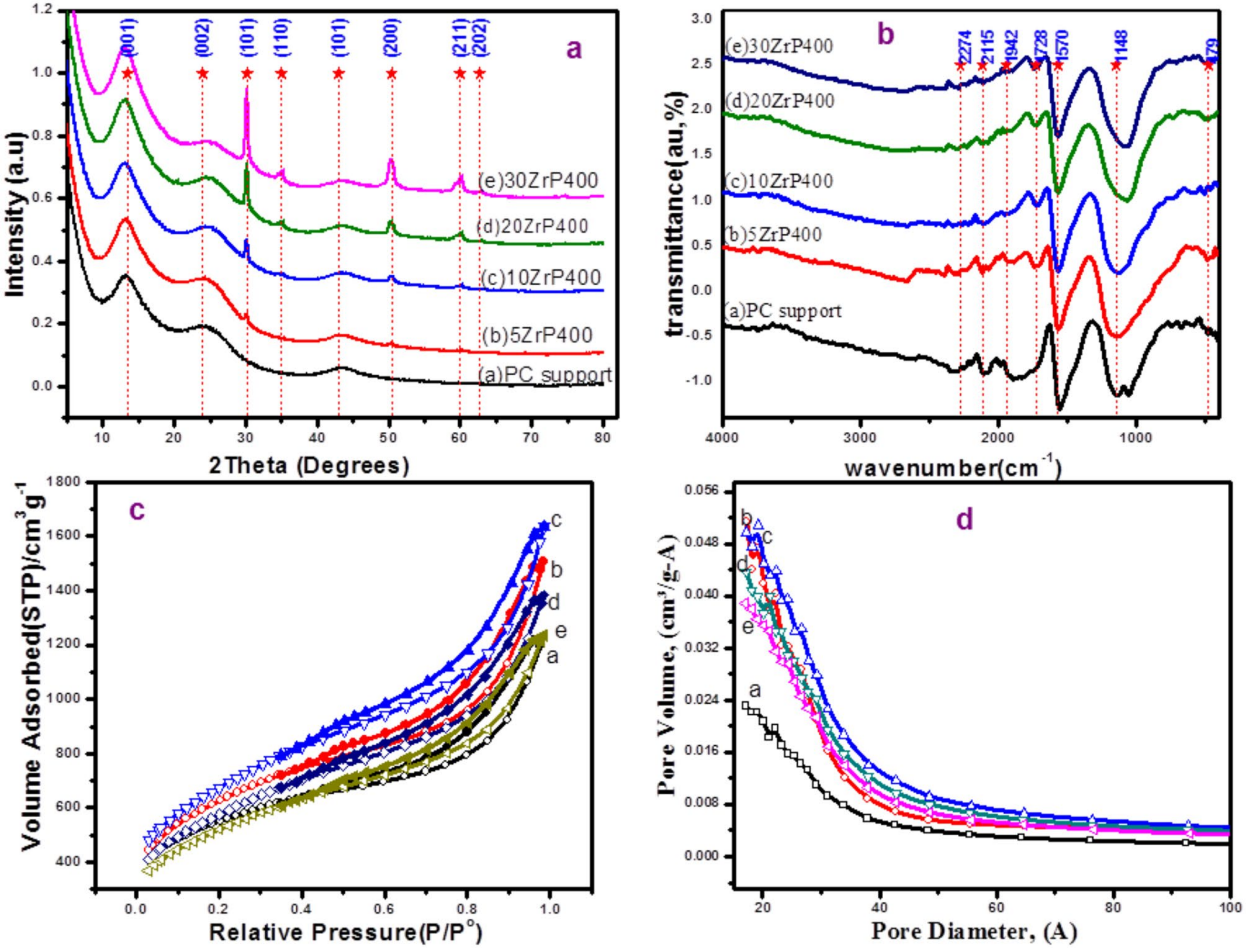
(**a**) XRD patterns, (**b**) FT-IR spectra, (**c**) N_2_ adsorption/desorption isotherms of ZrP400adsorbents, and (**d**) the corresponding pore size distribution of 5-30ZrP400 along with PC support

XRD for 5–30 ZrP400 nano composites annealed at 400 along with the corresponding blank PC biochar were shown in Fig. 1(a), respectively. Generally, all the calcined adsorbents with various loading ratios were influenced by the amorphous nature of graphite. Consequently, the patterns displayed extremely distinct broad peaks close to two theta, 24 and 43º, which correspond to the graphite structure’s (002) and (101) crystal planes at (2θ = 24.2° and 43.13°, File No., 75-1621) [14]. Thus, indicating the formation of nanosized amorphous graphite structure of few graphene layers. It is clear that the intensity of peak near 2θ = 24 decreased as a new peak at 2θ = 13.13 appears, which corresponding to (001) crystal plane of graphite oxide [60–62]. For supported materials 5–30%ZrO_2_, The (111), (200), (220), (311), and (331) was equivalent to crystal planes of cubic ZrO_2_ phase (PDF#49–1642) [63] with maintenance with the peak of graphite structure. Diffraction peaks appear at around 30.12^º^, 24.96º, 50.22º, 59, 74º, and 81.76◦. The intensity of the peak grew as the zirconia ratio increased, revealing the ZrO_2_ phase’s very distinct crystalline structure.

### ATR-FTIR spectroscopy

FTIR-ATR spectroscopy was utilized to analyze the surface functional groups of the PC biochar and the 5–30% ZrP400 series treated at 400 °C, as shown in Fig. 1 (b). The spectrum of the PC biochar exhibited characteristic peaks, including the stretching vibration of *ν*(C=O) at 1690 cm^− 1^, *ν(*C = C) at 1570 cm^− 1^ [64], *ν*(C−O) at 1144 cm^− 1^ [65, 66] and P−O−C in addition to P−O−P at 1080 cm^− 1^ [67]. Asymmetric stretching and bending modes of the phosphate ion were identified as the source of the absorption bands in the 1000–1100 cm^–1^ region and the band at 478 cm^–1^, respectively [68]. The ZrP400 supported materials exhibited the same distinctive functional groups as in the PC carbon substance. Additionally, in materials loaded with ZrO_2_, a band at 1723 cm^− 1^ corresponds to the stretching vibration modes of the OH bond and is linked to the chemisorption of hydroxide groups and water on the ZrO_2_ nanoparticles [69]. The Zr-O vibration mode is responsible for the bands seen at 480 and 580 cm^−1^, as indicated by the spectrum, confirming the creation of the ZrO_2_ structure [70, 71]. Further evidence that Zr (IV) sites are accessible for interactions with the phosphoric carbon matrix comes from the Zr-O-P bond, which was also responsible for the shift from 1130 cm^− 1^ for bare phosphoric carbon to 1050 cm^− 1^ for supported ZrP400 materials.

### Textural characteristics

The nitrogen adsorption/desorption isotherms forthe5-30ZrP400-supported adsorbents and those for the blank PC support are displayed in Fig. 1(c). Type *IV* isotherms were the categorization given to the isotherms based on the first IUPAC classification. Mesoporosity with a slit-shaped pattern was shown by the *H*_*3*_ hysteresis loops seen in the isotherms. For PC support, 5 and 10 ZrP400 supported adsorbents, respectively, the values of the total N_2_ adsorbed volume (*V*_*T*_) increased to 1.113, 2.021, and 2.205 ccm/g. *V*_*T*_ values decreased to 1.895 and 1.698 ccm/g for ZrO_2_PC ratios of 20 and 30, respectively. The enlargement of the slit-shaped hole brought about by the loading of a tiny quantity (5–10%) of zirconia phase into carbon matrix is responsible for the first rise. The following decline at the highest loading (20−30%) can be attributed to pore filling or blockage). Further textural details were calculated and cited in Table S1.

For PC support, a high surface area of *S*_*BET*_ = 1797 m^2^/g (*S*_*Ex*t_ = 1209, *S*_*mic*_ = 588 m^2^/g) was noted. The 5–10% ZrP400 supported adsorbents showed a higher surface area, with *S*_*BET*_ values of 2236 and 2412 m^2^/g (*S*_*Ext*_ values of 1857 and 2107, *S*_*mic*_ values of 379 and 305 m^2^/g). However, *S*_*BET*_ gradually and somewhat decreased (2056 and 1857 m^2^/g) as the zirconia loading was raised to 20 and 30%, respectively. Simultaneously, the values of *S*_*Ext*_ and *S*_*mic*_ were progressively reduced by augmenting the zirconia loading into PC support; refer to Table S1. Figure 1 (d), presents the pore width distribution (PWD) obtained from the 5–30 ZrP400 and blank PC support materials. The standard pore width values for the adsorbents, *W*_*P*_, were calculated using the average pore width (*4V*_*P*_*/S*_*BET*_) and BJH methods, as referenced in Table S1. The PWD was slightly enhanced in terms of pore volume and distribution range expanded in the order of 5 > 10 > 20 > 30% > PC support. This confirms successive insertion of ZrO_2_ phase in the slit-shaped pores of the PC biochar that lead to reduction of the pore volume.

### Surface properties of nano composite

**Fig. 2:**
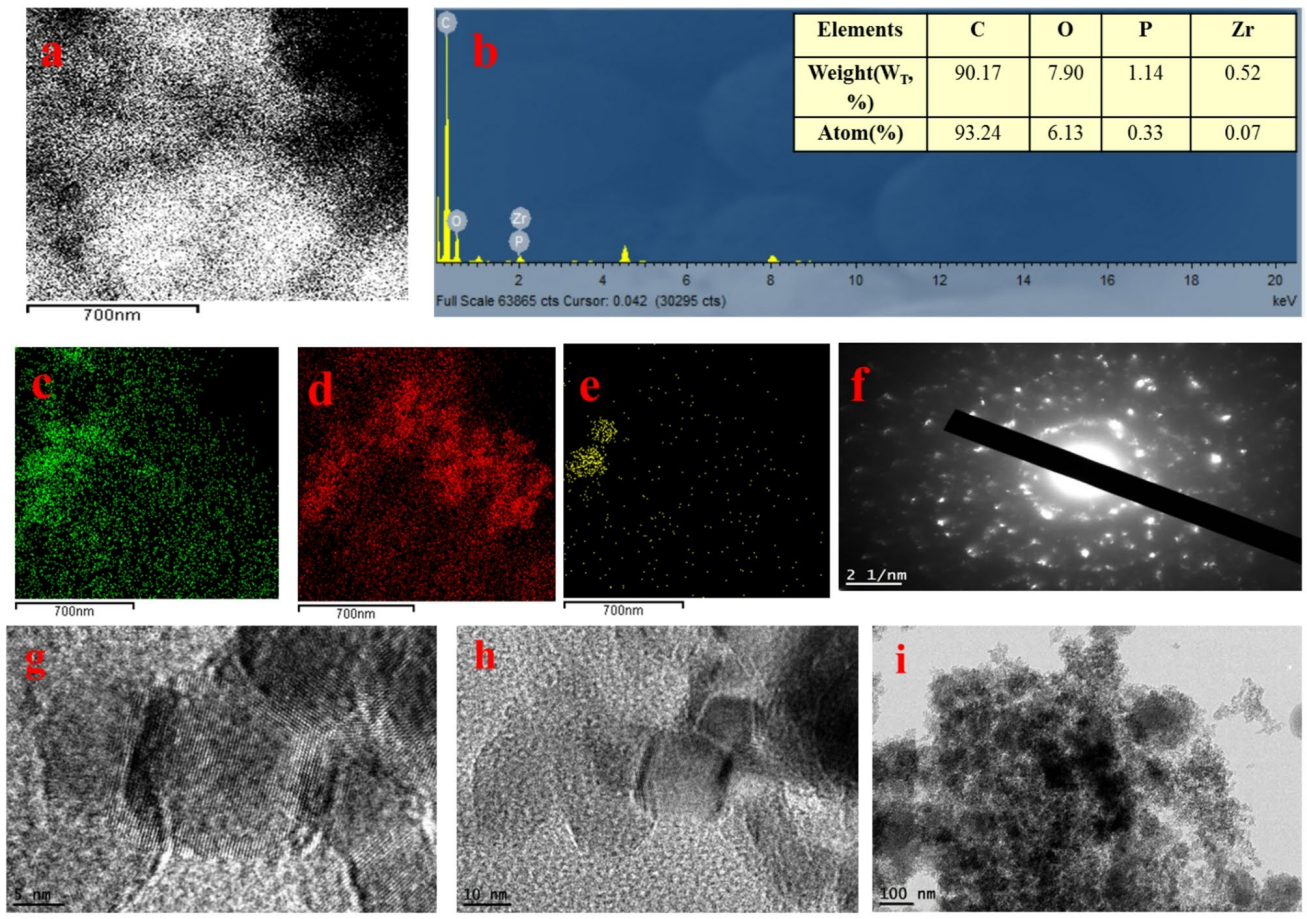
EDX mapping of 5ZrP400 adsorbent (**a**-**e**), HR-TEM, micrographs for 5ZrP400 adsorbent at different magnification (**g**-**i**), and electron diffraction (**f**)

**Fig. 3:**
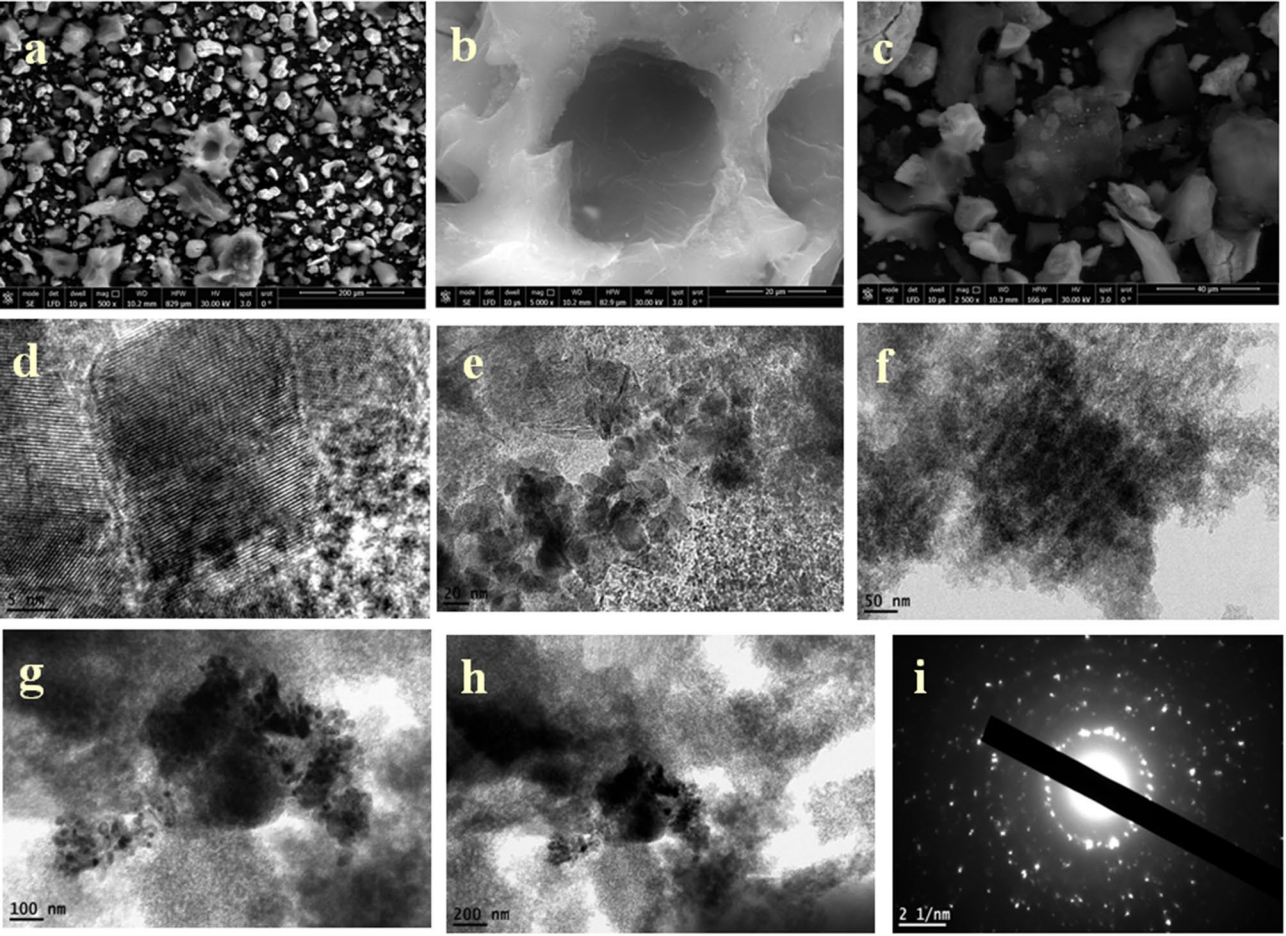
FESEM morphologies of 30ZrP400 adsorbent (**a**-**c**), HR-TEM, micrographs for 30ZrP400 adsorbent at different magnification (**d**-**h**), and electron diffraction (**i**)

**Fig. 4:**
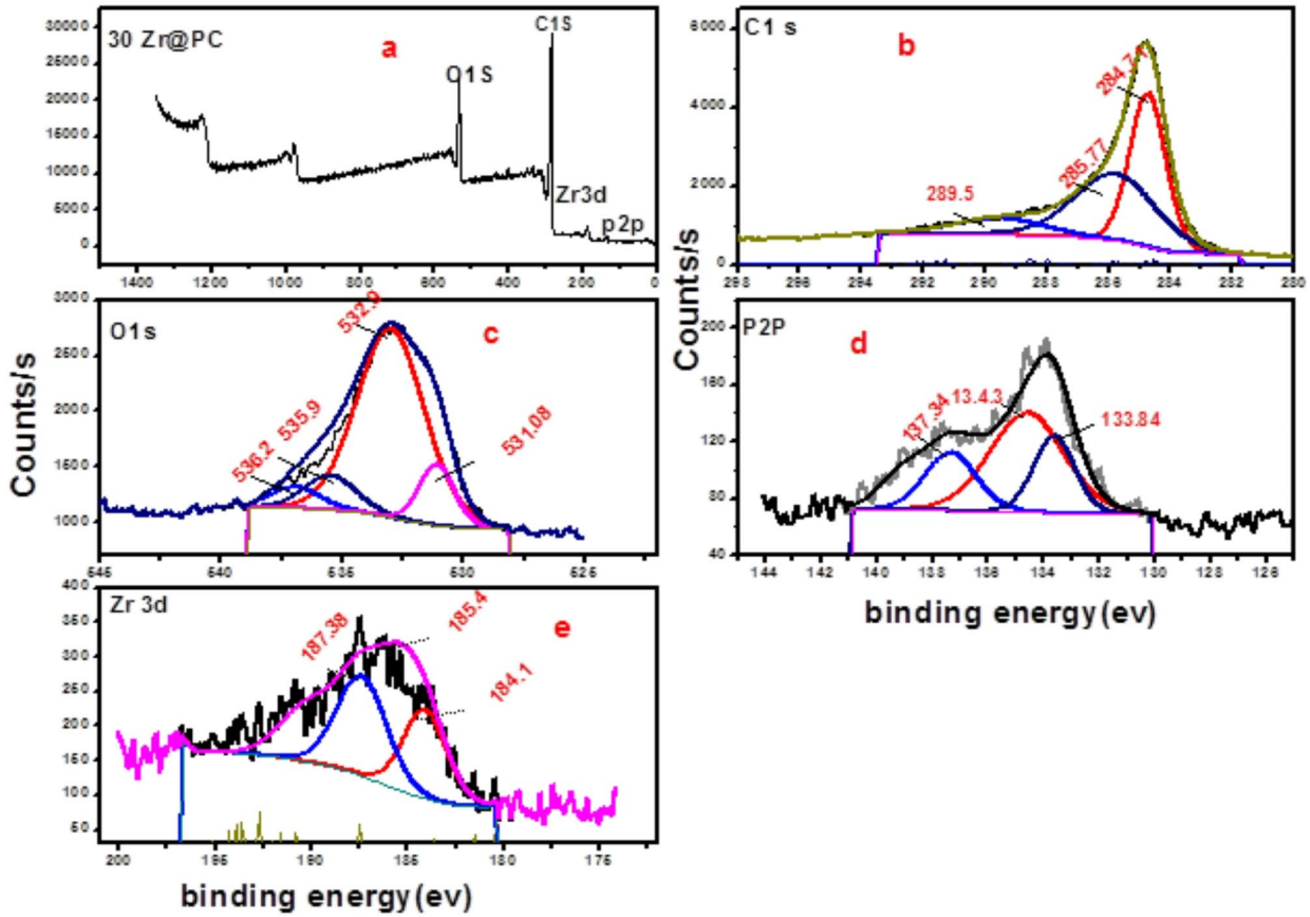
Extremely detailed XPS spectra of 30 ZrP400 (**a**) C1s. (**b**) O1s. (**c**) P2p. (**d**) and Zr3d. (**e**)

For the 5ZrPC400 material, elemental mapping using (EDX) was performed, as indicated in Fig. 2 (a-e). The EDX mapping results confirmed the presence of C, O, P, and Zr elements, indicating a highly uniform distribution of these elements throughout the carbon matrix (PC), with no bulk phase formation. This uniform distribution is essential for maintaining consistent adsorption behavior. High-resolution transmission electron microscopy (HRTEM) images of 5ZrP400 Fig. 2 (g-i) and 30ZrP400 materials Fig. 3 (d-h) at various magnifications revealed that the material exhibits porous characteristics, consisting of nanosized aggregates of plate-like amorphous particles smaller than 5 nm. Additionally, well-organized ZrO₂ nanoparticles were observed, revealing a lattice plane (111) of ZrO₂ with a d-spacing of approximately 0.316 nm corresponds to the distance between adjacent planes in the ZrO₂ lattice, a critical feature for providing active sites that may further enhance adsorption or facilitate chemical interactions with pollutants. Selected area electron diffraction (SAED) patterns of 5ZrP400 Fig. 2(f) and 30ZrP400 Fig. 3 (i) were also analyzed, where the amorphous zones displayed diffuse rings and the crystalline zones exhibited bright spots, confirming the crystallinity of ZrO₂. This indicates even further that the ZrO₂ nanoparticles were successfully prepared and dispersed on the phosphoric carbon matrix surface, in agreement with the XRD data. The very porous texture of 30ZrP400 with evenly dispersed ZrO₂ nanoparticles is revealed by (FE-SEM) micrographs, which are displayed at different magnifications in Fig. 3 (a-c). Using (XPS), the elemental compositions and different chemical states of 30ZrP400 were examined, as shown in Fig. 4. C, O, P, and Zr are present, according to the elementary survey Fig. 4(a).For C1s spectrum, the peaks at binding energies 284.4 and 284.6 eV were assigned to the *sp*^2^-bonded of (C=C) and *sp*^3^-bonded carbons (C−C), respectively. The п-п^*^ transition in aromatic rings is associated with the peak at 289.5 eV, while the carbonyl group is given the peak at 285.7 eV [72, 73]. for O1*s* spectrums the peak centered at 532.6 eV can be attributed to single bonded oxygen in C−OH, C–O–C and/or C–O–P, P–O–P linkages [16, 74]. Phosphate, metaphosphate, and P_2_O_5_ were identified as the sources of the peaks at 133.4, 134.4, and 136.8 eV in the XPS spectrum of P2p [75]. For Zr3d spectrum, exhibit two group peaks centering at ∼185 eV and ∼183 eV, corresponding to Zr3d_3/2_ and Zr3d_5/2_, respectively [76]. Furthermore, the high-resolution O1*s* spectra show only one broad peak that may be further deconvoluted into four peaks. The peak centered at 532.18 eV was identified as Zr-OH on the surface of 30ZrP400, while the peak centered at 531.07 eV corresponds to the oxygen present as Zr-O-Zr. A second peak, which confirms the addition of phosphorus, is located at 531.53 and 532.76 eV and corresponds to Zr-O-P and P-O-H, respectively [77].

### Batch-based adsorption

#### Influence of ZrO₂ ratios on amitriptyline adsorption

**Fig. 5:**
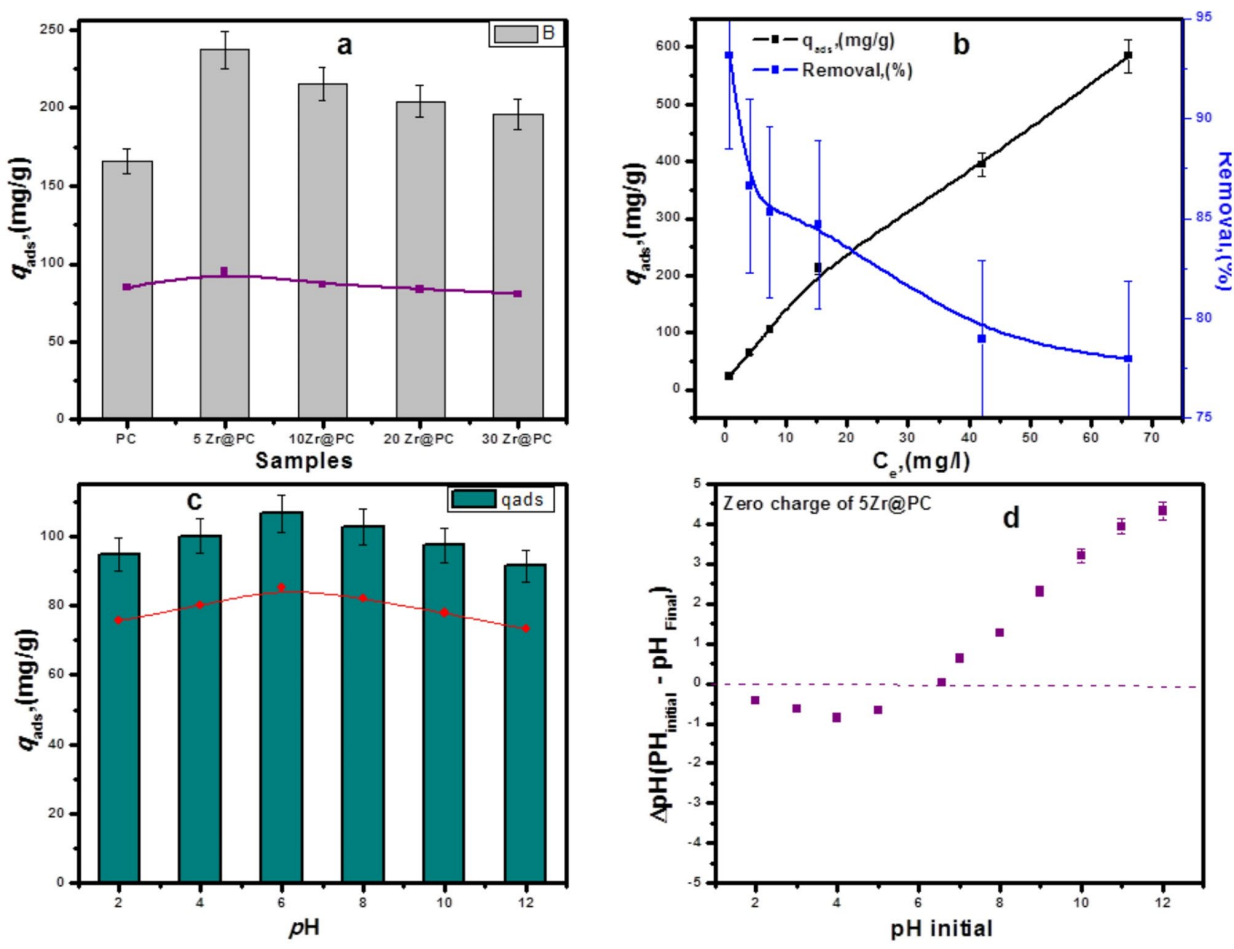
(**a**) Adsorption capacity and removal of AMT over PC support and 5-30ZrP400 adsorbent (**b**) Adsorption capacity and removal of the most active sample 5ZrP400 adsorbent, (**c**) effect of pH on the qads and the removal (%) of AMT and (**d**) Point of zero charge of 5ZrP400 adsorbent. Error bars represent the samples’ standard deviation

Phosphorous Carbon and supported ZrP400 calcined at 400 ºC is displayed at Fig. 5 (a). The results show adsorption capacity and removal (%) of amitriptyline over PC biochar and 5–30 ZrP400adsorbents, The highest adsorption capacity of amitriptyline was observed for 5 ZrP400 adsorbent reached to 237 mg/g with removal rate 94.5%, Furthermore, increasing the Zr-loading from 10 to 30% ZrO₂ led to a gradual decline in the adsorption capacity for AMT, decreasing from 215 mg/g to 195 mg/g, with the removal rate decreasing from 86 to 80%, respectively. To examine the impact of Zr content in phosphoric-acid-doped biochar on AMT removal, 5ZrP400 was tested at various AMT concentrations ranging from 10 to 300 mg/L. The results, shown in Fig. 5(b), the adsorption capacity was found to be 585 mg/g, achieving a 78% removal rate at an initial concentration of 400 mg/L. This adsorption performance indicates the presence of numerous dominant functional groups, which provided active sites for the adsorption of AMT.

#### The impact of pH on the adsorption of AMT

Figure 5(c) illustrates the impact of pH on the adsorption of the AMT drug using the 5 ZrP400 adsorbent across a pH range of 2 to 12. The highest adsorption efficiency (106.6 mg/g) was observed at a solution pH of 6. When the pH exceeded 6, the AMT removal rate rapidly declined with increasing pH. Below pH 6, the adsorbent surface became cationic due to the protonation effect of H^+^ ions. Conversely, above pH 6, the adsorbent surface became anionic due to the presence of OH^−^ ions. The effect of solution pH on q_ads_ and AMT removal over 5 ZrP400 is shown in Fig. 5(c). In the pH range of 2–5, strong repulsive forces exist between the cationic amitriptyline and the positively charged adsorbent surface. Additionally, there is intense competition between H + ions and amitriptyline cations for active sites on the 5 ZrP400 adsorbent, leading to a reduction in the adsorption potential of AMT [78, 79], However there is significant increase in adsorption capacity under neutral solution. At pH ˃ 6, the solution of amitriptyline converts to white precipitate of amitriptyline hydrochloride. Moreover, Adsorption capacity of AMT decreased to 91 mg/g. Therefore, the optimum pH for removal of AMT over 5 ZrP400 is 6(neutral solution). (PZC) of the 5 ZrP400 adsorbent was found to be at pH 6.52, which corresponds to a neutral surface charge, as illustrated in Fig. 5(d). Amitriptyline has a pKa of about 9.76, which indicates that because cationic amino groups predominate, it becomes positively charged at pH values lower than this value. As predicted, the electrostatic interaction between the positively charged amitriptyline cations and the anionic surface of the5ZrP400 adsorbent would be facilitated by a solution pH of between 6 and 9.76.

#### Impact of adsorbent dosage

A range of adsorbent dosages (0.200–6.000 g/L) at neutral pH was used, together with a fixed concentration of AMT (100 mg/L) to assess the AMT adsorption capacity (q_ads_) and removal percentage (%) of AMT. Fig. S1 showed that when the adsorbent dose rose, the 5ZrP400 biochar’s q_ads_ dropped from 277.5 to 15.6 mg/g at higher dosage, the adsorbent particles may aggregate, reducing the total effective surface sites available for adsorption. However, the 5 ZrP400 adsorbent’s extra adsorption sites improved AMT removal, increasing it from 55% at 0.200 g/L to 95.4% at 6.00 g/L. Consequently, 2 g/L is the ideal dosage to achieve equilibrium in the amount and rate of adsorption [80, 81].

#### Impact of AMT ratios

**Fig. 6:**
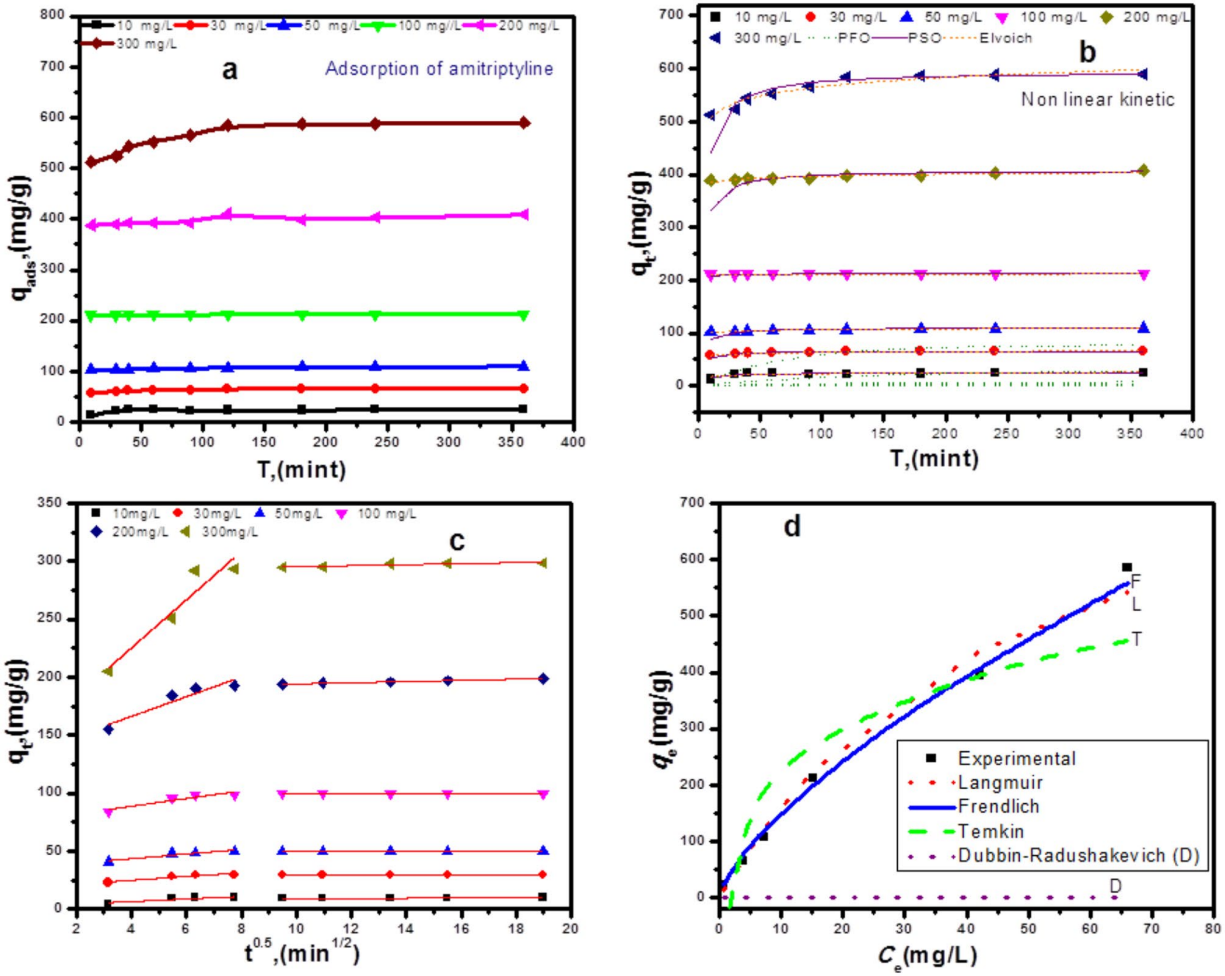
Impact of contact duration on 5ZrP400of AMT at different concentrations (**a**) Nonlinear PFO, PSO and Elovich, and (**b**) intra particle diffusion modeling (**c**) nonlinear Langmuir, Freundlich and Dubinin-Redushckevich (D-R) isothermal models (**d**)

AMT concentrations ranging from 10 to 300 mg/L, adsorbent dosages of 2.000 g/L, neutral pH, temperature of 35 ± 1 °C, and progressive durations of 10–360 min were used to study the adsorption rate. The results presented in Fig. 6 (a) show that when the adsorption period is extended, the adsorption efficiency gradually improves. AMT’s adsorption capacity on 5ZrP400 rises with the dynamic force of the concentration gradient.

#### Adsorption kinetic study

The mechanisms relating to 5ZrP400 and amitriptyline were examined through studies on adsorption kinetics characteristics. The experimental data was fitted with the pseudo-first-order [82], pseudo-second-order model [83], intra-particle diffusion model [84] and Elovich model [85]. To simplify the adsorption process and investigate the adsorption kinetics further. Several kinetic models were employed in both their linear and nonlinear forms to understand the adsorption kinetics and elucidate the mechanism. Figure S2 displays the linearized forms of the four dynamic kinetic models (a, b, c, and d) for amitriptyline (AMT) adsorption. Additionally, the nonlinear fits for the most active sample, 5ZrP400 are shown in Fig. 6 (b). The equations for each kinetic model in both linear and nonlinear forms are provided in Table S2.

The results indicate that the pseudo-second-order kinetic model (PSO), with an R² value close to 0.999, fits the data significantly better than the pseudo-first-order model (PFO), which has R² values ranging from 0.2045 to 0.9435. This suggests that the pseudo-second-order model more accurately describes the adsorption kinetics of amitriptyline on the adsorbent, reflecting chemical interactions [86]. The pseudo-second-order model’s predictions of maximum adsorption capacity (q_max, model_) are notably closer to the experimentally observed values (q_max, exp_), confirming that chemisorption is the dominant mechanism in the adsorption process with 5ZrP400 adsorbent.

The intra-particle diffusion model Fig. 6 (c) addresses the mass transfer of species from the solution bulk to the solid phase, identifying the rate-limiting steps in this process. The plots from this model revealed that the adsorption process is governed by multiple stages. Specifically, two linear segments were observed. The first segment corresponds to the diffusion of amitriptyline from the solution to the external surface of the 5ZrP400 adsorbent. The higher rate constant (K_ind_) for the initial stage indicates, this suggests that amitriptyline is rapidly adsorbed onto the external surface of the adsorbent. During the second stage, amitriptyline moves through the internal pores of the 5ZrP400 adsorbent. As the potential difference diminishes, the ions progressively diffuse further into the adsorbent material. This stage, which is slower than the first, is influenced by either pore diffusion or intra particle diffusion the movement of particles within the porous structure of the adsorbent [15, 87]. Additionally, the suitability of the Elovich model to describe the adsorption process was evaluated, and it yielded a high correlation coefficient (R² = 0.95), There is a significant correlation between the model predictions and the experimental adsorption data, suggesting the effectiveness of the model. (t-test method) was used for the statistical analysis. P-values were found to be less than 0.05 for the statistically significantly Linear and nonlinear PSO of AMT adsorption process in Table S4, whease P-values were found to be greater than 0.05 for the statistically significantly Linear and nonlinear PFO of AMT adsorption. The greater value of the t-statistic indicates there is a significant difference.

#### Adsorption isotherms

The equilibrium adsorption properties were fitted using Langmuir, Freundlich and Dubinin-Redushckevich (D-R) isothermal models provides. Langmuir [88] Freundlich [89], Temkin [90], and Dubinin-Radushkevich isotherm [91]. All models were examined. Earlier reports on the assumptions that underlie each model are available in [55, 80]. Illustrations are provided for the four isotherms (a, b, c, and d) in their linearized versions in Fig. S3, while nonlinear fits specifically for the most active sample, 5ZrP400, are presented in Fig. 6 (d). Detailed equations for both linear and nonlinear forms of each isotherm model can be found in Table S3. The summary includes parametric equations and constants derived from fitted data through nonlinear regression, outlining specific characteristics of the adsorption isotherms. The correlation coefficients (R²) for the four isothermal models rank as follows: R_F_² (0.9968) > R_L_² (0.7748) > R_T_² (0.7641) > R_D_² (0.4901). This indicates that the Freundlich model (R_F_² = 0.9968) best describes the kinetics of amitriptyline (AMT) adsorption on 5ZrP400. The Freundlich model’s application suggests non-uniform adsorption on a heterogeneous surface with multilayer attachment, supported by a Freundlich constant (K_F_) of 29.05 mg/g and an exponent (n) of 1.42, indicating preferential adsorption. Furthermore, higher concentrations of adsorbate result in greater effectiveness because of stronger contacts and enhanced accessibility between adsorbate and adsorbent, as demonstrated by the adsorption of AMT on 5ZrP400. The Langmuir adsorption model’s value of 1/n less than one suggests that AMT readily adsorbs onto the non-homogeneous surface of the adsorbent. The adsorption energy (E) of 34.094 KJ/mol for AMT on 5ZrP400 highlights the significant role of chemisorption in the adsorption process, indicating a chemical bonding mechanism.

#### Thermodynamics study

**Fig. 7:**
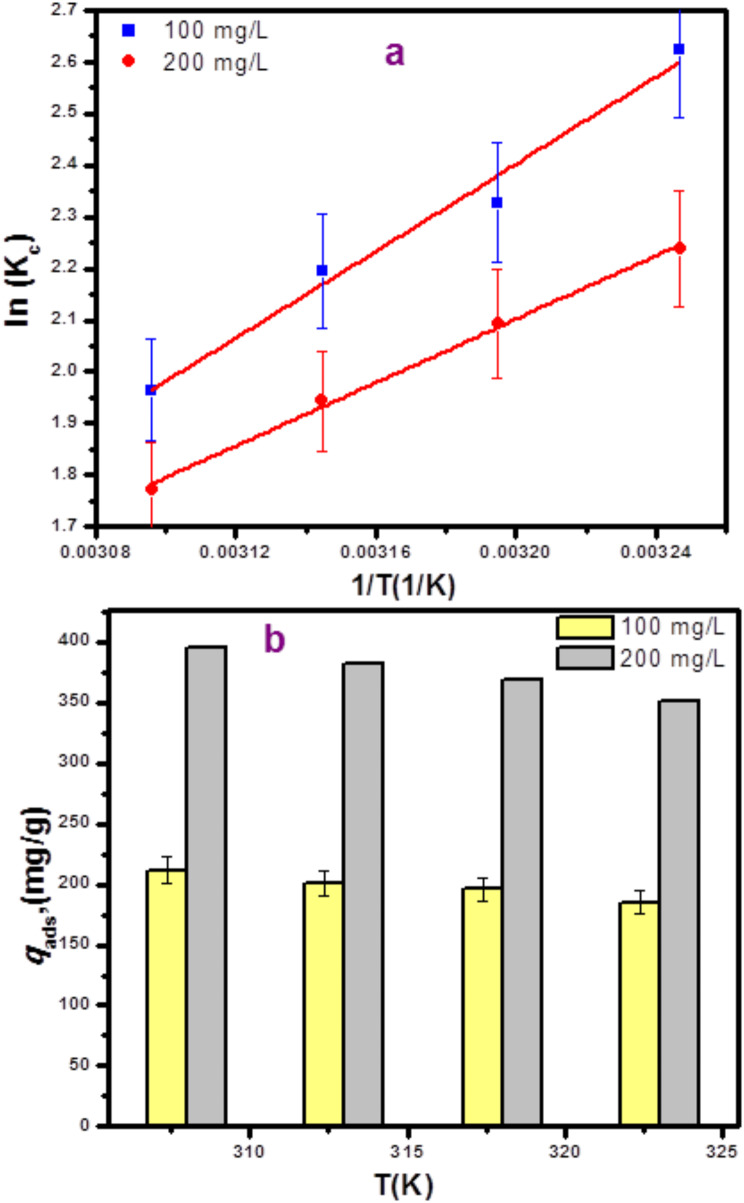
(**a**) Van’t Hoff plot of AMT adsorption on 5ZrP400 (initial concentration = 100,200 mg/L) (**b**) Temperature-related changes in adsorption capacity and Error bars represent the samples’ standard deviation

Impact of temperatures on AMT adsorption over 5ZrP400 adsorbent, were investigated at 35, 40, 45 and 50 °C. The results obtained with 5ZrP400 at two distinct beginning concentrations that were chosen, 100 and 200 mg/L are plotted. Figure 7 (a and b). From 35 to 50 °C, the adsorption capability of the 5ZrP400 adsorbent reduced as the temperature raised. This makes it clear that AMT drug adsorption onto AMT-ZrP400 is exothermic. Important metrics including ΔG°, ΔH°, and ΔS° were calculated; the results appear in Table 1. At the concentrations and temperatures under investigation, the ΔG° values were negative (-28.19 to -20.69 kJ/mol), suggesting that the amitriptyline-5ZrP400 adsorption system was spontaneous and thermodynamically favorable. The exothermic character of the adsorption process is indicated by the final value of ΔH°. The ΔH° value indicates physisorption or chemisorption adsorption process; it was observed that ΔH° is below 84 KJ.mol^− 1^. However, the positive value of ΔS° indicates a higher degree of unpredictability during the adsorption process [92].

**Table 1:** AMT elimination using a comparative analysis of several adsorbents

Adsorbent	pH	AMT C_o_(mg/L)	q_max_ (mg/g)	References
Polymer coated magnetic nano	8	100	47	[97]
Sonicated Ti_3_C_2_T_X_ MXene	7	0–22	241	[98]
Natural montmorillonite	9.4	500	276	[99]
Aminosilane-modified cellulose	7	100	404	[100]
3D boron-doped graphene composite	7	10–300	737	[79]
TiO_2_-activated carbon	7.4	10	823.9	[101]
Magnetic graphene-based material	9	50	91.84	[102]
ZrO_2_ phosphorus carbon ZrP400	7	10–300	585	This study

#### Regeneration and reuse of the Zrp400 adsorbent

**Fig. 8:**
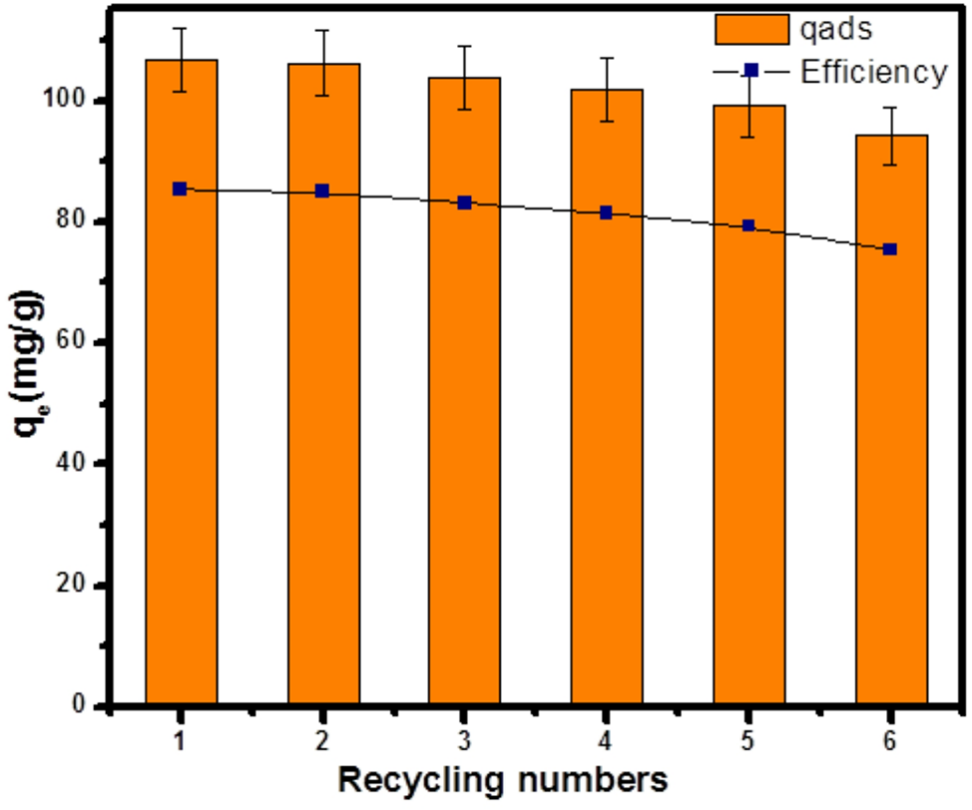
Cycling and regeneration of 5ZrP400 adsorbent. Error bars represent the samples’ standard deviation

The reusability of 5ZrP400 adsorbent was thoroughly evaluated through desorption experiments using an ethanol solution as the desorption agent. This approach is particularly important in assessing the sustainability and economic viability of the adsorbent, as the ability to regenerate and reuse the material multiple times reduces the need for fresh adsorbent. The reusability of 5ZrP400adsorbent was examined by employing a desorption agent consisting solely of ethanol solution due to its effectiveness in disrupting weak interactions (such as hydrogen bonds, π-π stacking, and electrostatic interactions) between the AMT molecules and the adsorbent surface. AMT-5ZrP400 was regenerated by desorption in an ethanol following each full adsorption, with the first adsorption amount acting as a baseline adsorption. According to Fig. 8, the percentage of AMT elimination varied from 85% in the first cycle to 75.3% in the fourth cycle, decreasing progressively through each cycle. This confirms that the 5ZrP400 adsorbent can be recovered and recycled again to remove AMT, demonstrating the adsorbent’s regenerative and reusable properties. It also shows that the adsorbent exhibits good regeneration and reusability [93].

#### EDX analysis and TEM of spent Zrp400adsorbent

**Fig. 9:**
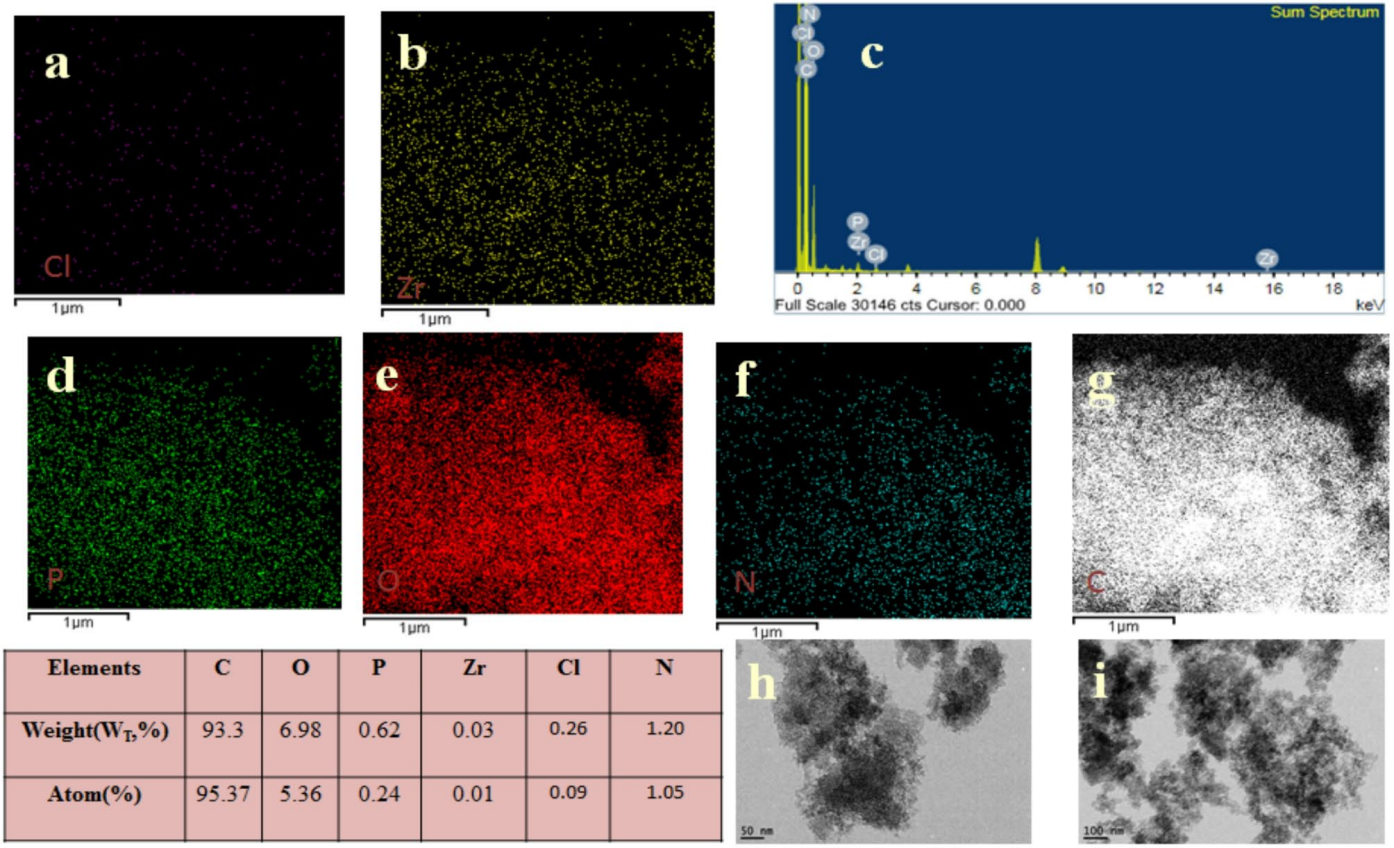
EDX mapping of AMT- 5ZrP400 adsorbent (**a**-**g**), HR-TEM, micrographs for AMT-5ZrP400 adsorbent at different magnification (**h**-**i**)

EDX elemental mapping Fig. 9 (a-g) confirmed the presence of the elements C, O, P, Zr, Cl, and N throughout the carbon matrix (PC) in a very uniform distribution, with no bulk phases generated. After AMT adsorption, there was a significant increase in the C content, indicating that AMT had been successfully adsorbed onto the surface of the 5 ZrP400 adsorbent. The appearance of additional peaks for Cl and N in the EDX spectrum confirmed the successful integration of the AMT molecule into the adsorbent structure. Compared to the pristine adsorbent, the elemental percentages of O, P, and Zr decreased, further suggesting that the addition of AMT onto the 5ZrP400 adsorbent was effective. The TEM images Fig. 9 (h-i) verified the porosity characteristics and fine morphology of the loaded Zr-carbon matrix, revealing amorphous aggregates of nanoscale graphite particles made of layers resembling graphene [14, 55, 80]. As a result, the EDX elemental mapping and TEM analysis provided strong evidence that the AMT molecule was successfully adsorbed onto the 5 ZrP400 adsorbent, with the adsorbent maintaining its porous and nanostructured characteristics after the adsorption process. The adsorption of the AMT drug resulted in a greater degree of aggregation of the 5ZrP400 support particles, but the overall morphological features remained similar to the pristine adsorbent. This increased aggregation likely enhanced the physisorption of the AMT drug onto the adsorbent surface. The FTIR-ATR spectra of 5ZrP400 before and after AMT adsorption are shown in Fig.S. Before adsorption, the spectrum (curve a) shows bands corresponding to the activated carbon framework (C, O, H) and the phosphorus-doped carbon framework (C, O, H, P), typical of materials treated with high H₃PO₄ ratios previous discuss in Sect. 3.2. After AMT adsorption, the spectrum of the spent 5ZrP400 reveals changes that confirm interactions between AMT and the adsorbent surface. The detection of a new band at 1249 cm^− 1^ was due to C–N stretching vibrations from amitriptyline. There is reduction in the band located at 1560 cm^− 1^ due to π–π interactions between the aromatic rings of AMT and graphitic carbon. The disappearance of the 2360 cm⁻¹ band indicates that the phosphorus-containing functional groups play a key role in the adsorption of AMT [94].

#### Mechanism of AMT adsorption into 5ZrP400

**Fig. 10:**
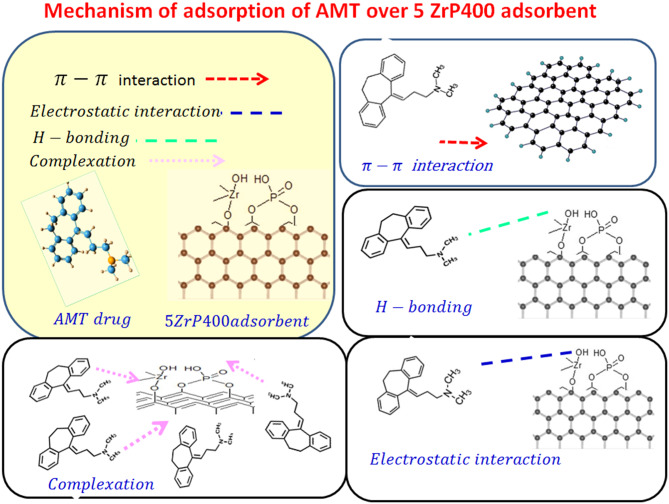
AMT adsorption mechanism by 5ZrP400 adsorbent

Van der Waals forces are the primary force behind physical adsorption, which is typically accompanied by chemical adsorption in the adsorption process. The 5ZrP400 adsorbent exhibits a high adsorption capacity, with adsorption energy (E) of approximately ~ 34.094 kJ/mol. Furthermore, the Freundlich model, which also shows that chemisorption controls adsorption efficiency, is a better fit for describing adsorption than the Langmuir model. As seen in Fig. 10 Chemisorption requires the formation of a chemical bond between the contaminant and the adsorbent. In AMT, the amino groups act as acceptors of hydrogen bonds. As a result, strong hydrogen bonds are formed between the 5ZrP400 adsorbent and AMT, strengthening the adsorption process even more. Moreover, the benzene ring and the heterocyclic ring in the AMT that contains nitrogen function as electron acceptors, whereas the OH group in the 5ZrP400 acts as an electron donor. As a result, π-π bonding interactions develop between AMT and 5ZrP400. Oxygen-containing functional groups, such as hydroxyl, phenolic, phosphate, and zirconia, were visible in the 5ZrP400 ATR-FTIR spectra. The improved and enhanced adsorption of AMT by surface complexation by oxygen bonding or weak bonds (van der Waals forces or electrostatic attraction) was attributed to the active site group (phosphate and zirconia) that was present at the surface of the adsorbent. Table 1 displays the previously reported adsorption capacities for various adsorbents. Among them, the 5ZrP400 adsorbent demonstrates a notably higher capacity for removing AMT from solution compared to the others.

#### An assessment of greene’s methodology

**Fig. 11:**
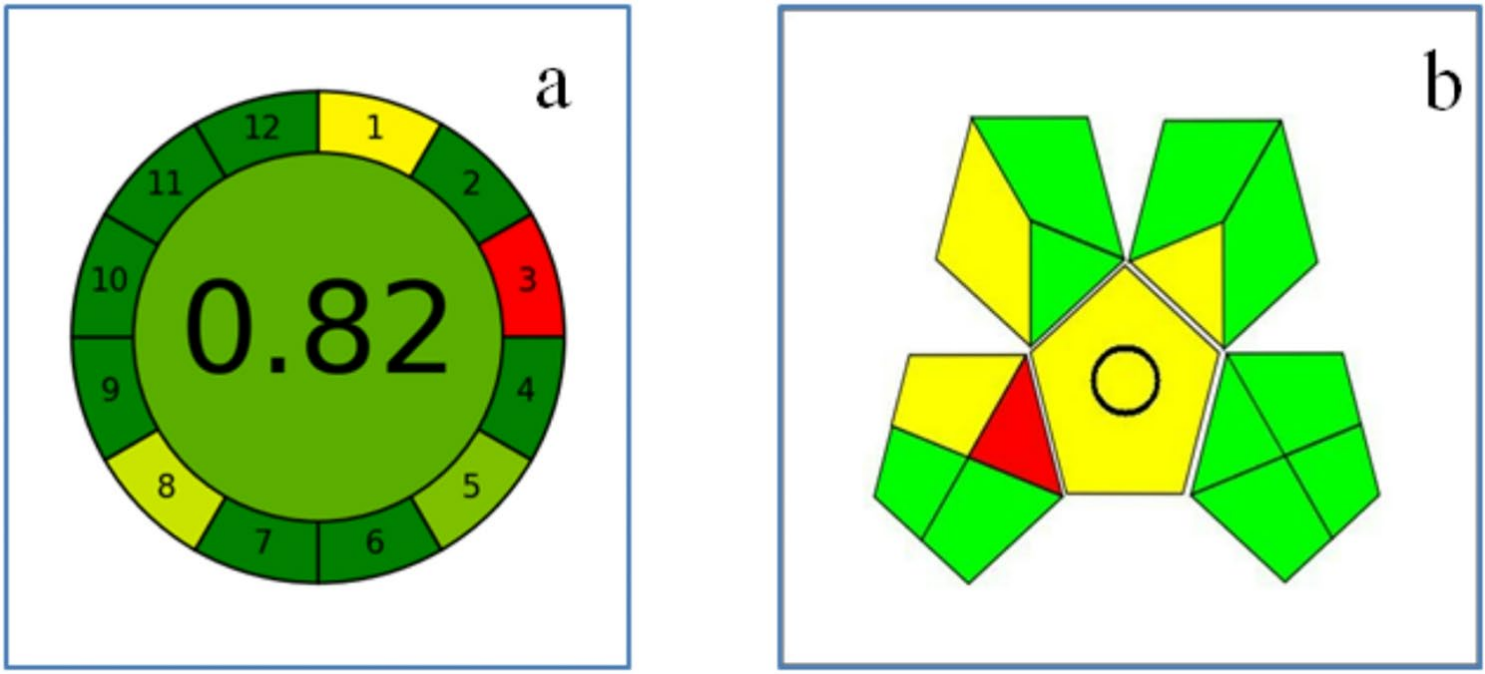
Evaluation of the greenness using AGREE (**a**) and GAPI (**b**)

The Green Analytical Procedure Index (GAPI) assesses the environmental effects of an analytical procedure from sample collection to final analysis [95]. The greenness of each stage is evaluated by GAPI using a color-coded pictogram (red, yellow, and green). Based on toxicity and solvent usage factors, the suggested approach in this assessment identified 4 yellow, 10 green, and 1 red zone. As a result, it was shown that the suggested approach had no impact on the environment. With a set of 12 criteria presented in a circular manner similar to a clock face and scores ranging from 0 to 1, AGREE Metrics [96] is a software tool that assesses the environmental and occupational risks related to an analytical procedure. Similar to the strategy described in another study, the central average score is a measure of the method’s environmental sustainability. Similar to the methodology described in a different study, the central average score functions as a gauge of the technique’s environmental sustainability. Figure 11 (a and b) illustrates, the positive results from the AGREE software indicate the use of a very successful green methodology. The outstanding outcome produced by the AGREE program indicates the application of a good green technique. The outstanding outcome produced by the AGREE program indicates the application of a good green technique.

## Conclusion

The synthesis of phosphorus biochar (PC) from sugarcane bagasse (SB), followed by the development of a zirconium-loaded PC nanocomposite (ZrP400), has demonstrated significant potential as a high-performance adsorbent for removing the tricyclic antidepressant amitriptyline from water. The study revealed that ZrP400 possesses a substantial surface area and porosity, providing abundant active sites for efficient adsorption. Under optimal conditions at a neutral pH, ZrP400 exhibited an impressive amitriptyline adsorption capacity of up to 585 mg/g with an adsorbent dosage of 2 g/L at 35 °C. The adsorption process adhered to the pseudo-second-order kinetic model and the Freundlich isotherm model, suggesting a combination of chemisorption and physisorption mechanisms. Additionally, the spent adsorbent could be effectively regenerated using ethanol, enhancing the process’s reusability and sustainability. Evaluations using GAPI and AGREE metrics further validated the environmental compatibility, practicality, and overall sustainability of the adsorption process, establishing ZrP400 as a promising solution for water treatment applications.

## Electronic supplementary material

Below is the link to the electronic supplementary material.

Supplementary Material 1

## Data Availability

The corresponding author can provide the data upon a reasonable request.
