# Supplementary material for: Environmental sustainable ZrO2 -phosphorous Biochar nano composite derived from sugarcane bagasse and their adsorption behavior of antidepressant drugs
Source: BMC Chem. 2025 Mar 14;19(1):68. doi: 10.1186/s13065-025-01430-4 (PMC11909959; doi:10.1186/s13065-025-01430-4)
Supplement: Supplementary file 1 — Supplementary Material 1 [file 13065_2025_1430_MOESM1_ESM.docx]

Environmental Sustainable ZrO_2_ -phosphorous biochar nano composite derived from sugarcane bagasse and their adsorption behavior of antidepressant drugs

Walaa A. Elhamdy

*Chemistry Department, Faculty of Science, Sohag University, P.O. Box 82524 Sohag, Egypt*

**Carbon material characterization**

X-ray diffraction patterns were obtained using a Phillips Spectrometer PW 2103/00, equipped with a Ni-filtered Cu Kα radiation source (λ = 1.5418 Å), to verify the structure and crystallinity of the materials. FTIR spectra were recorded with a Bruker Alpha FT-IR spectrometer (Germany) over a range of 400-4000 cm⁻¹. Nitrogen adsorption-desorption analyses were conducted at 196°C using a Micromeritics ASAP 2010 instrument (Micromeritics Corporation, USA). Samples were degassed at 200°C for 2 hours to a pressure of 0.1 Pa before measurement. The specific surface area (S_BET) was calculated using the Brunauer-Emmett-Teller (BET) equation [1]. The mesopore width distribution was determined using the Barrett-Joyner-Halenda (BJH) method [2] ,with all measurements and analyses conducted according to IUPAC standards [3]. Transmission electron microscopy (TEM) images were captured with a 100 kV JEOL 2000 device. Surface chemistry was analyzed using X-ray photoelectron spectroscopy (XPS, Thermo Fischer K-alpha) with monochromatic, micro-focused Al Kα radiation (1486.6 eV, spot size 400 μm). Field-emission scanning electron microscopy (FESEM) was performed with a QUANTA FEG 250 (Netherlands).

Table S1: Surface texture characteristics of PC support and ZrP400 composite materials.

| ***P*_W_(BJH)** | ***P*_W_(BET)** | ***P*_Meso_** | ***V*meso** | ***V*micro** | **V_T_** | ***S*micro** | ***S*Ext** | ***S*_BET_** |  |
| --- | --- | --- | --- | --- | --- | --- | --- | --- | --- |
| **(A^°^)** | **(A^°^)** | **(%)** | **(ccm/g)** | **(ccm/g)** | **(ccm/g)** | **(m^2^/g)** | **(m^2^/g)** | **(m^2^/g)** | **Sample** |
| 25 | 18 | 89.6 | 0.998 | 0.115 | 1.113 | 1209 | 588 | 1797 | **PC support** |
| 55.9 | 36.15 | 92.4 | 1.868 | 0.1526 | 2.0214 | 379 | 1857 | 2236 | **5ZrPC400** |
| 52.7 | 36.6 | 94.5 | 2.085 | 0.1206 | 2.2059 | 305 | 2107 | 2412 | **10ZrPC400** |
| 52.2 | 37 | 94 | 1.782 | 0.1134 | 1.8958 | 281 | 1775 | 2056 | **20ZrPC400** |
| 52 | 36.6 | 93.8 | 1.5932 | 0.10513 | 1.6983 | 262 | 1595 | 1857 | **30ZrPC400** |

Specific surface area (S*_BET_*), external surface area (S*_Ext_*), micropore area (S*_mic_*), total adsorbed volume (V*_T_*), micropore volume (V*_mic_*), mesopore volume (V*_meso_*), mesoporosity percentage (P*_Meso_*%) and pore width (P*_W_*).

Table S3: Parameters of Isotherm models for AMT removal by 5ZrP400

| **Isotherm type** | $\mathbf{linear form}$ | $\mathbf{nonlinear form}$ | **Parameters** |
| --- | --- | --- | --- |
| **Langmuir**  **Freundlich**  **Temkin**  **Dubinin-Radushevish** | $\frac{C_{e}}{q_{e}}=\frac{1}{q_{m b}}+\frac{1}{q_{m}} C_{e} Eq. \left( 14 \right)$ | $Q_{e}=\frac{q_{max\cdot K_{l}\cdot C_{e}}}{1+K_{l}\cdot C_{e}} Eq. \left( 15 \right)$ | $q_{m}=934.57$ $b=0.0209,$ $R_{l}=0.1375$ $R^{2}=0.7748$ |
|  | $logq_{e}=logk_{f}+\frac{1}{n} logC_{e} Eq. \left( 16 \right)$ | $Q_{e}=K_{f}\cdot C_{e}^{\frac{1}{n}} Eq. \left( 17 \right)$ | $\frac{1}{n}=0.7054,n=1.417$ $K_{f}=29.043$ $R^{2}=0.9963$ |
|  | $q_{e}=B_{T}lnK_{T}+B_{T}lnC_{e} Eq. \left( 18 \right)$ | $q_{e}=B_{T}\cdot\ln K_{T}\cdot C_{e} Eq. \left( 19 \right)$ | $B_{T}=117.86K_{T}=1.37, R^{2}=0.764$ |
|  | $\ln q_{e}=-B\cdot\varepsilon^{2}+\ln q_{s} Eq. \left( 20 \right)$  $\varepsilon=RT \ln\left( 1+\frac{1}{C_{e}} \right) Eq. \left( 21 \right)$ | $q_{e}=q_{s} \cdot e^{\left( -B\cdot\varepsilon^{2} \right)} Eq. \left( 22 \right)$ | *B=-7.011*10-8 E= 34.094 R^2^= 0.4901* |

Table S3: Kinetics parameters of the examined kinetic models for the adsorption of AMT on 5 ZrP400

| **C_o_**  **mg/L** | **Pseudo- first order model**  $\ln\left( q_{e-}q_{t} \right)=\ln q_{e -}K_{1}t \left( linear \right) Eq. \left( 7 \right)$  $q_{t}=q_{e}\left( 1-e^{-Kt} \right) \left( nonlinear \right)Eq.\left( 8 \right)$ | | | | **Pseudo- Second order model**  $\frac{t}{q_{t}}= \frac{1}{K_{2}q_{e}^{2}} + \frac{1}{q_{e}} \cdot t \left( linear \right) Eq. \left( 9 \right)$  $Q_{t}=\frac{{K_{2}\cdot Q_{e}^{2}\cdot t}}{1+K_{2}\cdot Q_{e}t} (nonlinear) Eq.(10)$ | | | |
| --- | --- | --- | --- | --- | --- | --- | --- | --- |
|  | ***q*_e_(mg/g)** | ***K*_1_** | ***R*^2^** |  | | ***q*_e_(mg/g)** | ***K*_2_** | ***R*^2^** |
| **10** | 2.34 | -0.0067 | 0.20455 |  |  | 24.94 | 0.00729 | 0.9983 |
| **30** | 5.19 | -0.0073 | 0.2714 |  |  | 65.78 | 0.00653 | 0.9999 |
| **50** | 8.18 | -0.0076 | 0.9923 |  |  | 109.89 | 0.00380 | 0.9999 |
| **100** | 2.054 | -0.0025 | 0.9078 |  |  | 212.76 | 0.0204 | 1 |
| **200** | 29.24 | -0.0085 | 0.8554 |  |  | 408.16 | 0.00105 | 0.9998 |
| **300** | 77.45 | -0.0149 | 0.9436 |  |  | 595.24 | 0.000485 | 0.9999 |

| **C_o_**  **mg/L** | **Intraparticle diffusion**  $q_{t}=K_{int}t^{1/2}+C Eq. (11)$ | | | | | |  | **Elvoich model**  $q_{t}=\frac{1}{\beta}\ln\left( \alpha\beta\right)+\frac{1}{\beta}\ln t \left( linear \right) Eq. \left( 12 \right)$  $q_{t}=\frac{1}{\beta}\ln\left( \alpha\beta_{t} \right) \left( nonlinear \right) Eq. (13)$ | | |
| --- | --- | --- | --- | --- | --- | --- | --- | --- | --- | --- |
|  | **1^st^ Stage** | | | **2^ed^ Stage** | | |  |  | | |
|  | ***K*_int_**  **(mg/g h^1/2^)** | ***C*** | ***R*^2^** | ***K*_int_ (mg/gh^1/2^)** | ***C*** | ***R*^2^** |  | ***α***  **mg g^-1^**  **min^-1^** | ***β*gmg^-1^** | ***R*^2^** |
| **10** | 1.1238 | 2.1104 | 0.77056 | 0.0756 | 8.475 | 0.9507 |  | 1.762 | 0.4113 | 0.4308 |
| **30** | 1.638 | 18.325 | 0.8376 | 0.00288 | 29.84 | 0.2981 |  | 3.78 | 0.40976 | 0.9069 |
| **50** | 1.986 | 35.677 | 0.80914 | -0.0118 | 50.08 | 0.4627 |  | 3.104 | 0.4633 | 0.9669 |
| **100** | 3.3195 | 35.677 | 0.81716 | 0.0626 | 98.89 | 0.6315 |  | 6.332 | 2.687 | 0.8704 |
| **200** | 8.445 | 132.49 | 0.8508 | 0.5247 | 188.8 | 0.9675 |  | 4.285 | 0.1904 | 0.8117 |
| **300** | 20.77 | 142.16 | 0.86577 | 0.4408 | 290.7 | 0.6871 |  | 3.0487 | 0.0396 | 0.9229 |

In above equations, q_e_ and q_t_ signify the amounts of Cr(VI) ions adsorbed per adsorbent mass (mg/g) at equilibrium and at time (h) time of adsorption while k_1_ (h^−1^), rate constant for pseudo-first order and k_2_ (g/mg.h), rate constants for pseudo- second-order kinetic models, respectively. Kint (mg g^−1^ h^−0.5^) and C (mg/g) are characteristic coefficient of intra particle diffusion. α (mg g^−1^ min^−1^) is initial adsorption rate, and β(g/mg) is desorption coefficient.

Table S4: Data obtained from t-test method for adsorption of AMT by 5ZrP400 adsorent(LinearPSO-nonlinearPSO, Linear PFO –non linear PFO).

**Table S5**: Thermodynamic parameter for AMT adsorption by 5ZrP400.

| **Concentration**  **mg/L** | **Temp**  **(ͦ K)**  **(KJ/** | **Δ*G* ͦ**  **(kJ/mol )** | **Δ*H* ^ͦ^**  **(kJ/ mol)** | **Δ*S* ^ͦ^**  **(kJ/molK^-1^)** |
| --- | --- | --- | --- | --- |
| **100** | 308  313  318  323 | -63.34  -63.79  -64.26  -64.72 | -34.99 | 92.011 |
| **200** | 308  313  318  323 | -45.25  -45.57  -45.89  -46.21 | -25.49 | 64.120 |

Fig.S1: effect of carbon dose on adsorption capacity and removal of AMT on 5 ZrP400 adsorbent. Error bars represent the samples' standard deviation.


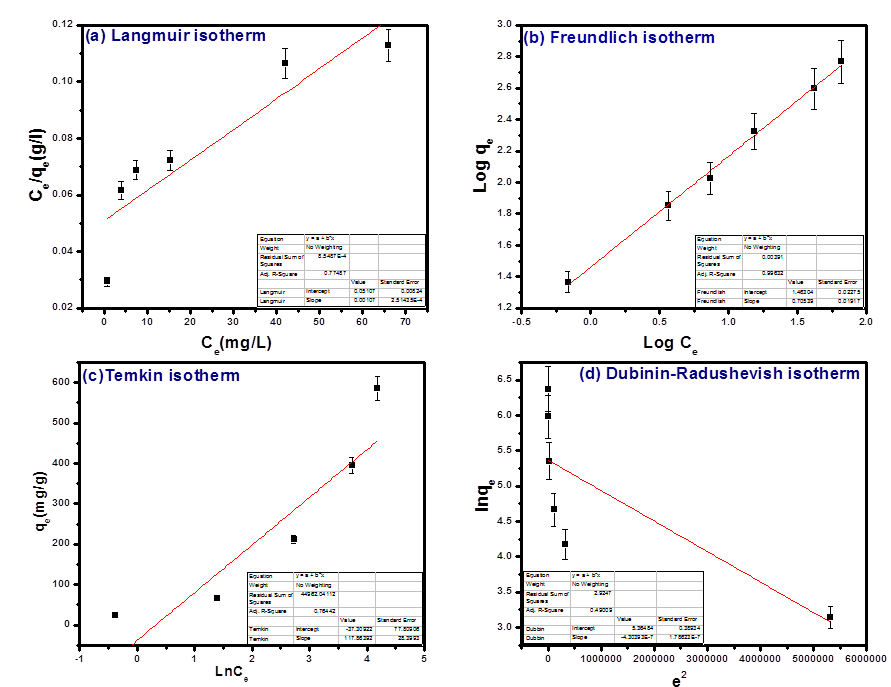


Fig.S3: Linear forms of the investigated adsorption isotherm models: the Langmuir (a), Freundlich (b), Dubbinin-Radushevish (c) and Temkin (d). Error bars represent the samples' standard deviation


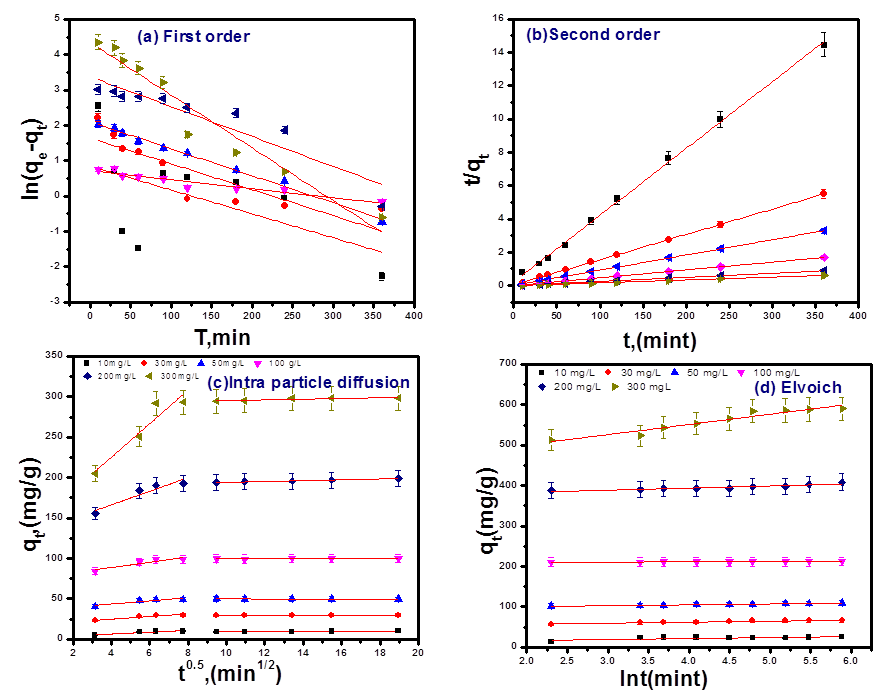


Fig.S4:The empirical adsorption of AMT on 5ZrP400 fitting with the different kinetic models pseudo first order (a), pseudo second order (b), Intra particle diffusion (c) and Elvoich models (d) at the indicated initial AMT concentrations 10-300 mg/L

Fig.S5: FTIR-ATR of ZrPP400 before (a) and after (b) adsorption of AMT.
